# Improved Therapeutic Efficacy of MT102, a New Anti-Inflammatory Agent, via a Self-Microemulsifying Drug Delivery System, in Ulcerative Colitis Mice

**DOI:** 10.3390/pharmaceutics15122720

**Published:** 2023-12-02

**Authors:** Kshitis Chandra Baral, Sang Hoon Lee, Jae Geun Song, Seong Hoon Jeong, Hyo-Kyung Han

**Affiliations:** College of Pharmacy, Dongguk University-Seoul, Goyang 10326, Republic of Korea

**Keywords:** solubility, inflammation, self-microemulsifying drug delivery system, oral efficacy

## Abstract

MT-102 is a new anti-inflammatory agent derived from *Juglans mandshurica* and *Isatis indigotica*. Its therapeutic potential is hindered by low aqueous solubility, impacting its in vivo efficacy. Therefore, this study aimed to develop a self-microemulsifying drug delivery system (SMEDDS) for MT-102 to enhance its oral efficacy in treating ulcerative colitis. Solubility assessment in different oils, surfactants, and cosurfactants led to a SMEDDS formulation of MT-102 using Capmul MCM, Tween 80, and propylene glycol. Based on a pseudoternary phase diagram, the optimal SMEDDS composition was selected, which consisted of 15% Capmul MCM, 42.5% Tween 80, and 42.5% propylene glycol. The resulting optimized SMEDDS (SMEDDS-F1) exhibited a narrow size distribution (177.5 ± 2.80 nm) and high indirubin content (275 ± 5.58 µg/g, a biomarker). Across an acidic to neutral pH range, SMEDDS-F1 showed rapid and extensive indirubin release, with dissolution rates approximately 15-fold higher than pure MT-102. Furthermore, oral administration of SMEDDS-F1 effectively mitigated inflammatory progression and symptoms in a mouse model of ulcerative colitis, whereas pure MT-102 was ineffective. SMEDDS-F1 minimized body weight loss (less than 5%) without any significant change in colon length and the morphology of colonic tissues, compared to those of the healthy control group. In addition, oral administration of SMEDDS-F1 significantly inhibited the secretion of pro-inflammatory cytokines such as IL-6 and TNF-α. In conclusion, the SMEDDS-F1 formulation employing Capmul MCM, Tween 80, and propylene glycol (15:42.5:42.5, *w*/*w*) enhances the solubility and therapeutic efficacy of MT-102.

## 1. Introduction

Inflammatory bowel diseases (IBDs) are chronic immune disorders in the intestine, such as Crohn’s disease and ulcerative colitis [1]. These conditions involve persistent, recurring inflammation that is either transmural and granulomatous or in the mucosa and submucosa regions of the gastrointestinal (GI) tract [2]. Although the precise causes and pathophysiological mechanisms of IBD are still undefined, it is believed that a combination of genetic predisposition, host immune responses, environmental factors, and intestinal microbial flora contribute to the pathogenesis [3]. Key factors in IBD pathogenesis also include various anti-inflammatory and pro-inflammatory cytokines, including tumor necrosis factor (TNF)-α, interleukin (IL)-4, and IL-6 [4]. Conventional treatment options, including 5-aminosalicylate-based compounds, corticosteroids, antibodies, and immunomodulatory and immunosuppressive agents, are primarily aimed at symptomatic relief, with surgical resection as an additional option when required [5]. However, there remains a gap in available treatments owing to the limited efficacy and adverse effects associated with current medications.

MT-102 is a natural extract derived from *Isatis indigotica* and *Juglans mandshurica*, exhibiting a good anti-inflammatory effect [6]. Given its poor aqueous solubility, MT-102 exhibits low oral bioavailability [6]. Thus, effective solubilization of MT-102 is essential for enhancing its oral efficacy. Among various formulation strategies, self-microemulsifying drug delivery systems (SMEDDSs) serve as a highly effective approach for improving the aqueous solubility of hydrophobic drugs [7,8]. In a SMEDDS, the drug is integrated into an isotropic mixture of oil, surfactant, and either a cosurfactant or a cosolvent [9]. Upon exposure to GI fluids, these mixtures spontaneously form fine oil-in-water (*o*/*w*) microemulsions in aid of GI motility [7]. In a SMEDDS, the pre-concentrated drug exists in a liquid state, circumventing the rate-limiting dissolution step that solid drugs typically encounter prior to intestinal absorption [10]. Furthermore, SMEDDSs are thermodynamically stable and transparent microemulsions, featuring droplet sizes between 100 and 250 nm [11]. This results in an expansive interfacial surface area favorable for drug partitioning between the oil phase and the GI fluid. SMEDDSs also enhance lymphatic drug transport, thereby circumventing hepatic first-pass metabolism and enhancing oral bioavailability [12]. Therefore, SMEDDSs extensively improve the oral bioavailability of hydrophobic drugs and phytochemicals. For example, Zhang et al. [13] successfully enhanced the oral bioavailability and anti-hyperuricemic activity of isoliquiritigenin using a SMEDDS that included ethyl oleate (oil), Tween 80 (surfactant), and PEG 400 (cosurfactant). Similarly, Liu et al. [14] showed that a SMEDDS formulation of ferulic acid (FA) effectively reduced renal clearance and increased the oral bioavailability of FA. Moreover, the FA-loaded SMEDDS augmented the distribution of FA in the brain, thus enhancing its hypnotic effect in a mouse model of insomnia [14].

**Figure 1:**
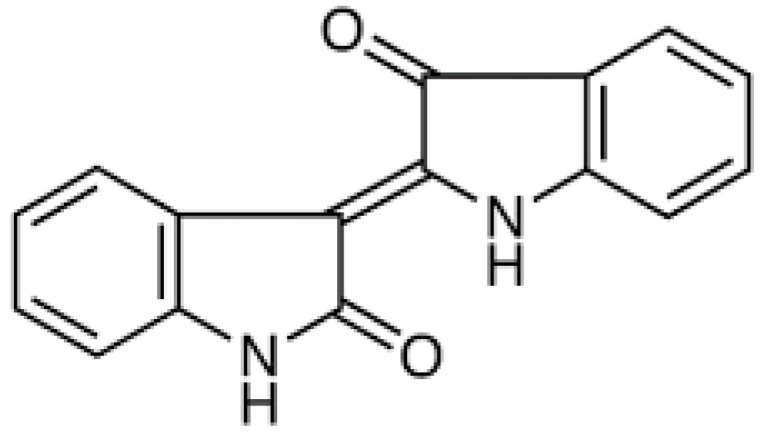
Indirubin structure.

In the present study, a SMEDDS of MT-102 was developed to enhance its solubility and oral efficacy in treating ulcerative colitis. The composition of the SMEDDS was optimized, and its effect on the therapeutic efficacy of MT-102 was assessed using a colitis-induced mouse model, with indirubin (Figure 1) serving as a biomarker.

## 2. Materials and Methods

### 2.1. Materials

MT-102 was obtained from MTHERA PHARMA (Seoul, Republic of Korea). Olive oil, corn oil, and soybean oil were purchased from Daejung Chemicals and Materials Co., Ltd. (Gyeonggi-Do, Republic of Korea). Transcutol HP, Masine CC, Peceol, Capryol 90, Labrafil M 1944 CS, Labrafil M 2125CS, and Labrasol were purchased from Gattefossé (Saint-Priest, Lyon, France). Kolliphor RH 40, Kolliphor HS 15, and Kolliphor ELP were obtained from BASF (Ludwigshafen, Germany). Capmul PG8, Captex 355, and Capmul MCM C8 were provided by ABITEC Corporation (Janesville, WI, USA). Tween 80, ethanol, polyethylene glycol 200 (PEG 200), propylene glycol, and 6-methoxyflavone were purchased from Sigma Aldrich (St. Louis, MO, USA). Dextran sulfate sodium salt (colitis grade) was purchased from MP Biomedicals (Solon, OH, USA). All solvents used for HPLC analysis were purchased from Merck KGaA (Darmstadt, Germany).

### 2.2. Selection of SMEDDS Components

#### 2.2.1. Solubility Assessment

The solubility of MT-102 was assessed in different oils, surfactants, and cosurfactants. Excess MT-102 was added to each vehicle, followed by vortex mixing for 2 min. The mixtures were then incubated at 37 °C in a shaking water bath for 72 h. After incubation, the mixtures were centrifuged at 17,000× *g* for 10 min, and the supernatants were collected and filtered through 0.45 µm membrane filters. Concentrations of indirubin in the filtrates were quantified using HPLC.

#### 2.2.2. Transmittance and the Efficiency of Emulsification 

For assessing the emulsification efficiency of different surfactants with a chosen oil (Capmul MCM), 250 µL of each surfactant was mixed with an equal volume of Capmul MCM and heated for 2 min at 40 °C. The resulting mixture was then diluted with water by a factor of 100 and inverted multiple times to achieve a clear emulsion. The number of inversions needed for clear emulsion formation was recorded. Following a 2 h stabilization period, transmittance (%) at 638 nm was measured using a UV–Vis spectrophotometer [7].

Similarly, the emulsification ability of cosurfactants was assessed with the selected oil and surfactant. First, 20 µL of each cosurfactant was mixed with 40 µL of the chosen surfactant and 60 µL of oil. This mixture was then heated for 2 min at 40 °C under vigorous stirring. Upon diluting the mixture 100-fold with distilled water, the number of flask inversions required for a transparent emulsion formation and the transmittance (%) were measured using the aforementioned method.

### 2.3. Phase Diagram

Building on the solubility assessment in Section 2.2, Capmul MCM, Tween 80, and propylene glycol were chosen as the oil, surfactant, and cosurfactant, respectively, for formulating a SMEDDS. To identify the self-emulsifying region and estimate the concentrations of the SMEDDS components, pseudoternary phase diagrams were constructed employing a water titration technique at 37 °C. For each diagram, a homogeneous mixture (S_mix_) of the chosen surfactant and cosurfactant was formulated at varying weight ratios (1:1, 1:2, 2:1, 1:3, and 3:1). This S_mix_ was then blended with the selected oil at various weight ratios in individual glass vials, resulting in a transparent and homogeneous mixture of oil and S_mix_. Each of these mixtures was titrated with water while being gently stirred (50 rpm) at 37 °C. The formation of spontaneous emulsions and the phase clarity were visually assessed. During the water titration process, any transitions in physical states from transparent to turbid, or the converse, were monitored. The phase diagrams were subsequently generated using SigmaPlot^®^ (Systat Software Inc., San Jose, CA, USA) to indicate the self-emulsifying regions.

### 2.4. Fabrication of MT-102-Loaded SMEDDSs

Utilizing the pseudoternary phase diagram, a set of SMEDDS formulations containing MT-102 were fabricated, varying the weight ratios of the selected oil, surfactant, and cosurfactant. In every formulation, the MT-102 content was maintained at a constant 20% (*w*/*w*). The oil, surfactant, and cosurfactant were mixed homogenously with vigorous stirring. Subsequently, MT-102 was introduced into the mixture and vortexed to yield a clear solution. The resulting SMEDDS formulations were stored at ambient temperature until further analysis.

### 2.5. In Vitro Characterization of MT-102-Loaded SMEDDSs

#### 2.5.1. Droplet Size, Zeta Potential, and Shape

Each SMEDDS formulation was diluted with water by a factor of 100. Dynamic light scattering (DLS) was conducted using a Zetasizer Nano-ZS90 (Malvern Instruments, Malvern, UK) to ascertain the average droplet size and zeta potential of the resultant microemulsion. The polydispersity index (PDI) was also determined to assess the distribution of particle sizes.

Transmission electron microscopy (TEM) analysis was conducted to examine the morphological properties of the selected SMEDDSs at the Korean Basic Science Institute (Daegu, Republic of Korea).

#### 2.5.2. Self-Emulsification Efficiency and Robustness to Dilution 

The prepared SMEDDSs were diluted by a factor of 10, 100, and 1000 using water, 0.1 mol/L HCl, or pH 6.8 phosphate buffer, and stirred at 37 °C at 100 rpm. Each diluted sample was observed for phase separation or precipitation during 24 h storage at room temperature. 

For the measurement of self-emulsification time, each SMEDDS formulation (1 mL) was combined with water (500 mL) at 37 °C and stirred at 100 rpm. The time needed to establish a transparent emulsion system upon dilution was recorded.

#### 2.5.3. Stability 

The impacts of centrifugation, temperature, and freeze–thaw cycles on the physical state of the SMEDDSs were evaluated to assess the thermodynamic stability of the SMEDDSs. A selected SMEDDS formulation was diluted 1:100 with water and placed through centrifugation at 5000 rpm for 30 min. The SMEDDS formulation was also subjected to six heat and cool cycles, alternating between 48 h storage at 4 °C and 40 °C. Additionally, the SMEDDS formulation underwent three freeze–thaw cycles, alternating between 48 h storage at −20 °C and 25 °C. Any change in the physical appearance or sign of phase separation was monitored at each cycle.

#### 2.5.4. In Vitro Drug Release 

In vitro drug release studies of pure MT-102 and the optimized SMEDDS (SMEDDS-F1) were conducted at 37 ± 0.5 °C with stirring at 100 rpm. Each formulation containing 50 mg of MT-102 was enclosed in hard gelatin capsules and submerged in dissolution media at pH values ranging from 1.2 to 6.8 or in water. At each time point, samples were collected, and an equal volume of fresh medium was introduced into the vessel to sustain a constant dissolution medium volume. The collected samples underwent filtration through a membrane filter (a pore size: 0.45 µm). The released indirubin concentration was quantified by HPLC analysis.

In vitro drug release profiles were also examined in the simulated intestinal fluids (FaSSIF (fasted-state simulated intestinal fluid) and FeSSIF (fed-state simulated intestinal fluid)). FaSSIF was composed of 0.2 mmol/L lecithin, 3 mmol/L sodium taurocholate, 19.12 mmol/L maleic acid, 68.62 mmol/L sodium chloride, and 34.8 mmol/L sodium hydroxide. FeSSIF was composed of 2 mmol/L lecithin, 10 mmol/L sodium taurocholate, 55.02 mmol/L maleic acid, 125.5 mmol/L sodium chloride, 81.65 mmol/L sodium hydroxide, 5 mmol/L glyceryl monooleate, and 0.8 mmol/L sodium oleate [15,16,17]. The pH was regulated to 6.5 for FaSSIF and 5.8 for FeSSIF. In vitro drug release studies of pure MT-102 and the SMEDDS in the simulated intestinal fluids were performed at 37 ± 0.5 °C with stirring at 100 rpm, following the same procedures as described above.

### 2.6. In Vivo Efficacy in Ulcerative Colitis-Induced Mice

The oral efficacy of the MT102-loaded SMEDDS (SMEDDS-F1) was evaluated in dextran sulfate sodium (DSS)-induced colitis mice and compared to that of pure MT-102. The experimental protocol was approved by the Institutional Animal Care and Use Committee in Dongguk University (IACUC-2022-006-01). C57BL/6 mice (8 weeks old, 23–25 g) were acquired from Orient Bio Co., Ltd. (Seongnam, Republic of Korea). Prior to the study, the mice were acclimated for one week in a house facility. The mice were then randomly allocated into four experimental groups (*n* = 4 per group): healthy control, colitis control, MT-102 treatment, and SMEDDS-F1 treatment groups. Colonic inflammation was initiated by providing the mice with drinking water containing 1.5% (*w*/*v*) DSS for a 7-day period. Subsequently, each group received a daily oral dose of either PBS (200 mL), MT-102 (300 mg/kg), or SMEDDS-F1 (equivalent to 300 mg/kg of MT-102) for eight consecutive days (Days 0–7). On Day 7, the DSS-containing water was substituted with normal tap water for an additional two days. Throughout the study, the healthy control group was given normal tap water. On Day 9, all mice were euthanized, and their colons were harvested for histological analysis and therapeutic efficacy evaluation. Monitoring of body weight, stool consistency, and occurrence of blood in the stool or anal region took place daily. Furthermore, the disease activity index (DAI) was computed based on parameters including weight loss, stool consistency, and presence of blood.

### 2.7. Histological Analysis and Pro-Inflammatory Cytokine Secretion

For the histological examination of extracted colonic tissues, hematoxylin–eosin (H&E) staining was employed. Initially, the tissues were fixed in a 4% paraformaldehyde solution before being embedded in paraffin. These samples were then stained using H&E and imaged with an Eclipse Ti-U inverted microscope (Nikon, Tokyo, Japan). The colitis severity was assessed by examining mucosal characteristics, including mucosal edema, epithelial damage, and the presence of inflammatory cells.

Pro-inflammatory cytokines such as IL-6 and TNF-α in the colonic tissues were quantified using ELISA. After homogenizing the tissues in RIPA buffer supplemented with protease inhibitors, samples were centrifuged at 13,000× *g* for 10 min at 4 °C. Concentrations of IL-6 and TNF-α in the resulting supernatant were determined using ELISA kits (Invitrogen, Waltham, MA, USA), in accordance with the manufacturer’s guidelines.

### 2.8. HPLC Assay

Indirubin concentrations in the samples were determined using an HPLC system (Perkin Elmer series 200, Waltham, MA, USA). Samples were eluted through a C_18_ column (4.6 × 150 mm, 5 µm; Phenomenex, Torrance, CA, USA) at 30 °C. The isocratic mobile phase was a methanol–water mixture (70:30, *v*/*v*), and the flow rate was 1.0 mL/min with a detection wavelength of 289 nm. The lower limit of quantification (LLOQ) for indirubin was 0.1 μg/mL, which was based on a signal-to-noise ratio of 10:1 and met the acceptable criteria (accuracy and precision) within ±20%. A linear calibration curve (*r*^2^ = 0.999) was generated over a concentration range of 0.1–50 µg/mL, using 6-methoxy flavone as an internal standard. The coefficient of variation (CV%) was less than 2.79%, and the accuracy was within ±12% for all concentrations.

### 2.9. Statistical Analysis

All data are expressed as the mean ± standard deviation (SD). Statistical analysis was conducted using one-way ANOVA followed by Dunnett’s test. 

## 3. Results and Discussion

### 3.1. Screening of SMEDDS Components

**Figure 2:**
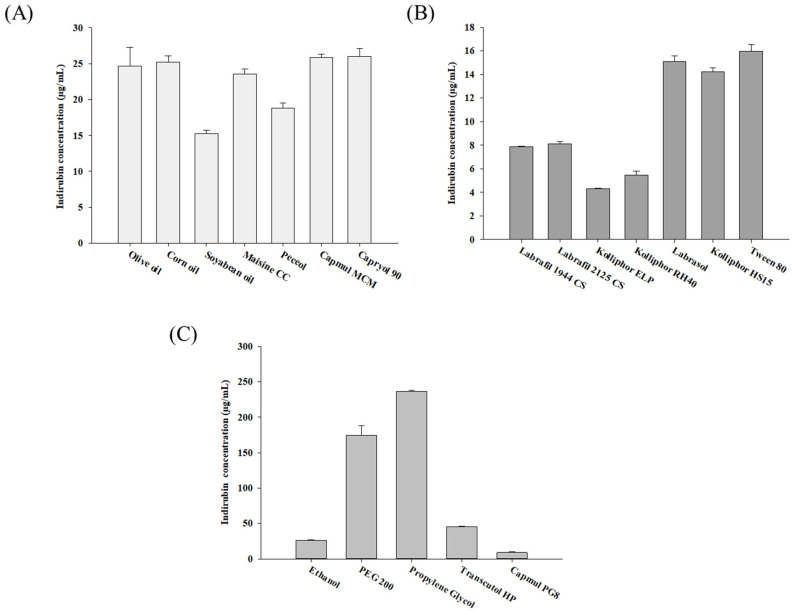
Indirubin concentration released from MT-102 in various oils (**A**), surfactants (**B**), and cosurfactants (**C**) (mean ± SD, *n* = 3).

The constituents of the formulation, including oil, surfactant, and cosurfactant, were selected to optimize the solubility enhancement of MT-102 in individual components for the development of a ternary SMEDDS. Within a SMEDDS designed for poorly water-soluble drugs, the lipid phase is the main factor affecting both drug solubility and the prevention of drug precipitation upon dilution. Furthermore, lipid components facilitate micelle formation and enhance lymphatic drug transport, thereby improving the intestinal absorption of poorly soluble drugs [18]. Owing to the influence of the physicochemical properties of oils, such as the molecular structure, triglyceride chain length, and degree of saturation, on the solubilization and emulsification processes [12], the solubility of MT-102 in various oils was assessed. The assessment involved determining the concentrations of indirubin, a marker compound, released from MT-102. As shown in Figure 2A, five oils exhibited similarly high indirubin concentrations, ranging from 24 to 26 µg/mL. Among these, Capmul MCM, composed of mono-diglycerides of medium-chain fatty acids, was selected as the oil phase for SMEDDS preparation due to its superior miscibility with surfactant and cosurfactant compared to other oils. In addition, medium-chain fatty acids are more rapidly digested in the gastric phase due to high gastric lipase selectivity, resulting in faster drug absorption [19,20]. 

For surfactant selection, the emphasis was on enhancing the solubility and emulsification efficiency of MT-102. Surfactants with high HLB values are particularly beneficial for instantaneously forming oil-in-water emulsions (*o*/*w*) [12]. Thus, surfactants with an HLB greater than 10 were examined in this study. Among the surfactants tested, Tween 80 (HLB: 15) was chosen as the non-ionic surfactant, exhibiting a high indirubin concentration (15.96 ± 0.59 µg/mL) (Figure 2B). Although Labrasol, Kolliphor HS15, and Tween 80 all showed a similarly high solubilizing effect, Tween 80 offered higher transmittance upon dilution and more efficient emulsification, requiring fewer flask inversions (Table 1 and Figure 2B).

Since transiently negative interfacial tension is rarely achieved with a single surfactant, SMEDDSs often require the addition of cosurfactants [21]. Cosurfactants featuring medium-chain-length alcohols (C3-C8) are commonly employed because they enhance the elasticity of the interfacial film between dispersed droplets and the external phase, thereby facilitating spontaneous microemulsion formation [12]. As shown in Figure 2C and Table 1, propylene glycol achieved the highest indirubin concentration (236.52 ± 1.72 µg/mL) along with good emulsification efficiency.

Taken together, Capmul MCM, Tween 80, and propylene glycol were selected as the formulation constituents for the SMEDDS designed for MT-102.

### 3.2. Construction of a Pseudoternary Phase Diagram

**Figure 3:**
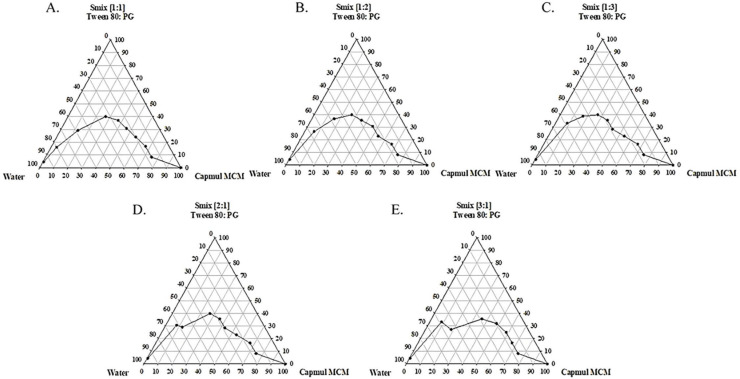
Pseudoternary phase diagram with oil, water, and S_mix_. Oil: Capmul MCM; S_mix_: mixture of surfactant (Tween 80) and cosurfactant (propylene glycol, PG) at various ratios.

Since surfactants and cosurfactants serve as a mechanical barrier against the coalescence of emulsion droplets and thus improve the thermodynamic stability of SMEDDSs [22], the ratio of oil to S_mix_ has a great impact on the formation of SMEDDSs. To delineate the self-microemulsifying regions, pseudoternary phase diagrams were constructed for systems containing Capmul MCM oil and S_mix_ at various surfactant-to-cosurfactant ratios (Tween 80 and propylene glycol), in the absence of any drug, as illustrated in Figure 3. The diagram revealed self-microemulsifying regions where dilution resulted in the formation of clear and transparent emulsions. As depicted in Figure 3, an S_mix_ (ratio of Tween 80 to propylene glycol at 1:1) encompassed 43% of the total diagram area, suggesting its high self-emulsification efficiency. Furthermore, this S_mix_ ratio (1:1) also showed the highest transmittance level (90.6%) in comparison to other S_mix_ ratios, which ranged from 69.6% to 81.5%. As a result, an S_mix_ ratio of 1:1 was selected for preparing the MT-102-loaded SMEDDS. 

### 3.3. Fabrication and In Vitro Characterization of the MT-102-Loaded SMEDDSs

#### 3.3.1. Preparation of Drug-Loaded SMEDDSs

To determine the optimal mixing ratio for the chosen components, drug-loaded SMEDDSs were formulated by varying the ratio of oil to S_mix_ at 1:1. When the ratio of oil to S_mix_ exceeded 15%, the average droplet sizes in the resulting microemulsions exhibited an increase, accompanied by poor emulsification efficiency (Table S1 in Supplementary File). Droplet size is a critical factor that influences both the rate and extent of drug release, as well as the lipolysis process, and ultimately affects drug absorption. Generally, smaller droplets yield a larger interfacial surface area, thereby enhancing the diffusion of emulsion droplets across the unstirred water layer and facilitating lymphatic drug transport. Of the formulations evaluated, SMEDDS-F1, consisting of Capmul MCM, Tween 80, and propylene glycol in a 15:42.5:42.5 (*w*/*w*) ratio, rapidly formed a microemulsion upon dispersion in water. It exhibited a small droplet size (177.5 ± 2.80 nm) with a narrow distribution and high transmittance (>90%) (Table 2). This formulation also displayed a negative charge, which likely aids in stabilizing the emulsion droplets against coalescence through electrostatic repulsion. Therefore, SMEDDS-F1 was chosen for additional characterization, encompassing thermodynamic stability, robustness to dilution, and drug release profiles.

#### 3.3.2. Thermodynamic Stability and Robustness to Dilution 

The stability of emulsion droplets can be notably influenced by thermal and mechanical stresses. To assess this, thermodynamic stability studies were carried out on SMEDDS-F1 under various stress conditions. The results verified the stability of the reconstituted microemulsions, which exhibited neither precipitation, phase separation, nor cloudiness following centrifugation, heat–cool cycles, and freeze–thaw cycles. Furthermore, during these stress studies, SMEDDS-F1 maintained a narrow particle size distribution, surface charge, and high indirubin content (Table 3).

Upon dilution with water, 0.1 mol/L HCl, or pH 6.8 phosphate buffer, SMEDDS-F1 preserved optical clarity without phase separation or precipitation. The average droplet size tends to decrease upon dilution, maybe due to the reduced agglomeration of particles at lower concentrations. These results suggest its potential for maintaining stability in the gastrointestinal tract (Table 4).

#### 3.3.3. Morphology

**Figure 4:**
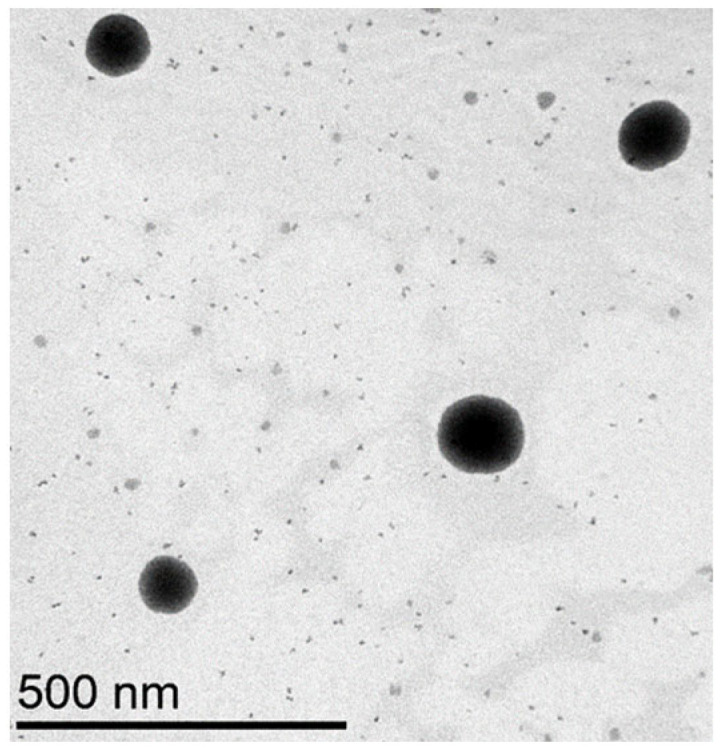
TEM image of SMEDDS-F1.

TEM analysis was conducted to examine the morphology of SMEDDS-F1. As illustrated in Figure 4, SMEDDS-F1 possesses a spherical morphology without evidence of aggregation. It is noteworthy that the droplet size observed through TEM was smaller compared to the average hydrodynamic diameter assessed by DLS. This discrepancy likely arises because the hydrodynamic diameter measured by DLS incorporates not only the particle core but also any stabilizers and ions bound to the particle surface and the layer of solvation.

#### 3.3.4. In Vitro Drug Release 

**Figure 5:**
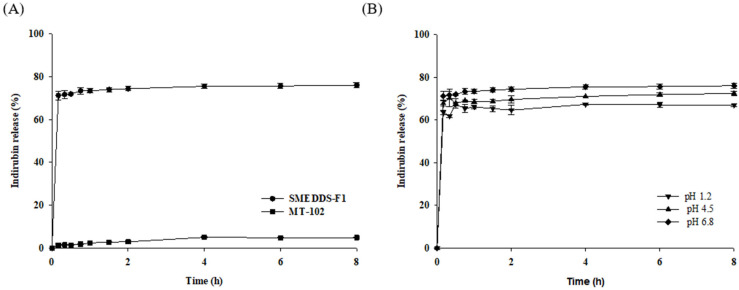
In vitro dissolution profiles of SMEDDS-F1 (mean ± SD, *n* = 3). (**A**) Indirubin release from SMEDDS-F1 and pure MT-102 in water; (**B**) Indirubin release from SMEDDS-F1 under various pH conditions.

The in vitro drug dissolution of SMEDDS-F1 and pure MT-102 was examined over a pH range of 1.2–6.8, using indirubin as a marker compound. As shown in Figure 5A, the SMEDDS-F1 formulation substantially elevated both the rate and extent of indirubin dissolution in water. Specifically, it achieved an indirubin dissolution of approximately 76% within the first hour, in contrast to the negligible release from pure MT-102 (<5%). This substantial increase in indirubin release by SMEDDS-F1 can be attributed to its rapid formation of isotropic emulsions when exposed to aqueous conditions. Moreover, indirubin release from SMEDDS-F1 was not influenced by pH and remained consistently high over the pH 1.2 to pH 6.8, implying effective drug release throughout the GI tract (Figure 5B).

**Figure 6:**
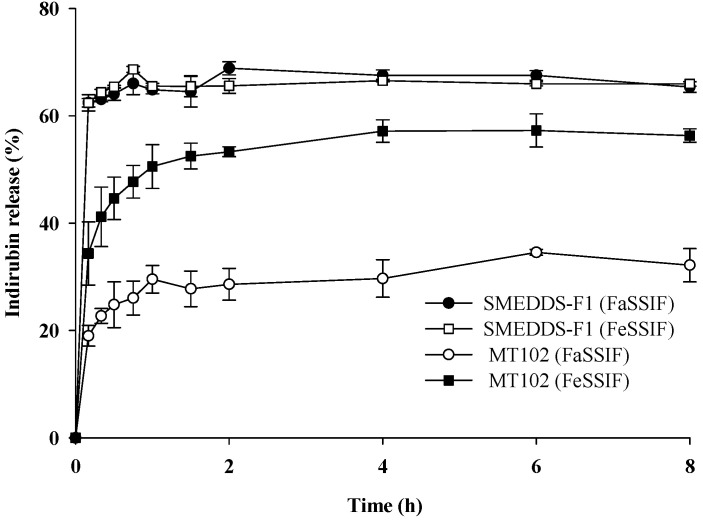
Dissolution profiles of the SMEDDS-F1 and pure MT-102 in FaSSIF and FeSSIF (mean ± SD, *n* = 3).

Given the influence of food intake on drug dissolution in the GI tract, drug release profiles of SMEDDS-F1 and pure MT-102 were examined using simulated intestinal fluids (FeSSIF and FaSSIF) to emulate fed and fasted states. As summarized in Figure 6, indirubin dissolution from pure MT-102 was notably elevated under fed conditions compared to fasted conditions. This observation can be attributed to increased concentrations of bile salts and phospholipids in the fed state. Functioning as surfactants, phospholipids and bile salts could facilitate the solubilization of hydrophobic drugs into micelles [23]. Furthermore, lipid digestion components such as glyceryl monooleate and sodium oleate present in FeSSIF contribute to the solubilization of lipophilic drugs. In contrast, SMEDDS-F1 demonstrated consistently high drug release profiles under both fed and fasted conditions (Figure 6). Compared to pure MT-102, the SMEDDS-F1 formulation achieved accelerated and significantly higher indirubin dissolution in both FeSSIF and FaSSIF. These findings suggest that SMEDDS-F1 is likely to mitigate the impact of food intake on drug absorption. 

### 3.4. In Vivo Efficacy Studies in DSS-Induced Colitis Mice

**Figure 7:**
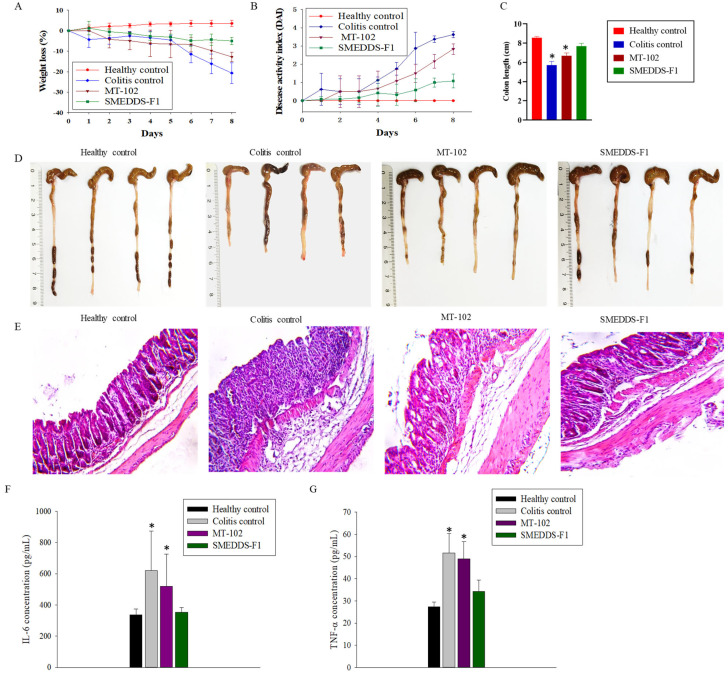
In vivo efficacy studies of SMEDDS-F1 in ulcerative colitis-induced mice. Efficacy was evaluated by the loss of body weight (**A**), disease activity index (**B**), colon length (**C**,**D**), H&E staining of colonic tissues (×40) (**E**), secretion of IL-6 (**F**), and TNF-α (**G**). Data are expressed as mean ± SD (*n* = 4). * *p* < 0.01 compared with the healthy control group.

The anti-inflammatory effects of the orally administered SMEDDS-F1 formulation and pure MT-102 were assessed in ulcerative colitis-induced mice by monitoring the severity of colitis, changes in body weight and colon length, histopathological evaluations, and the secretion of pro-inflammatory cytokines. The therapeutic efficacy of pure MT-102 was minimal in colitis mice, possibly due to the low oral absorption of the poorly soluble MT-102 (Figure 7). Conversely, the SMEDDS-F1 formulation demonstrated superior protective effects against inflammatory progression compared to pure MT-102. Specifically, while the colitis control group displayed a body weight loss of approximately 20.6%, the SMEDDS-F1 treatment group exhibited minimal loss, less than 5% (Figure 7A). Moreover, a significant improvement in the DAI was observed following oral administration of the SMEDDS-F1 formulation (Figure 7B). Unlike the colitis control and MT-102 treatment groups, the SMEDDS-F1 treatment group did not show symptoms such as diarrhea and blood in stools. In addition, no significant difference in colon length was noted between the SMEDDS-F1 treatment group and the healthy control group. In contrast, significant reductions in colon length were evident in both the colitis control and MT-102 treatment groups (Figure 7C,D). Histological examinations of colonic tissues further corroborated the therapeutic efficacy of the SMEDDS-F1 formulation. As depicted in Figure 7E, the morphology of tissues in the SMEDDS-F1 treatment group closely resembled that of the healthy control group, with an absence of significant microscopic inflammation markers such as epithelial erosion, interstitial edema, and infiltration of inflammatory cells.

Given the role of pro-inflammatory cytokines such as IL-6 and TNF-α in mediating colonic tissue damage, their production was assessed using ELISA. As shown in Figure 7F,G, oral administration of SMEDDS-F1 effectively reduced the secretion of these inflammatory cytokines. Previous studies have reported that indirubin, a biomarker of MT-102, inhibits the production of pro-inflammatory cytokines, thereby mitigating inflammatory responses and potentially ameliorating colitis symptoms [24,25]. For example, Gao et al. [24] demonstrated that indirubin treatment (10 mg/kg) for 7 days suppressed the loss of body weight with significantly alleviated mucosal injury and inflammatory symptoms. They also observed a significant decrease in the secretion of cytokines such as TNF-α, IFN-γ, and IL-2 by indirubin treatment. Additionally, indirubin has been shown to modulate immune responses by regulating the activity of immune cells implicated in colitis, including T cells and macrophages, restoring immune balance and diminishing excessive inflammation [24,25]. Tokuyasu et al. [26] demonstrated that oral intake of indirubin in a diet could improve the DSS-induced ulcerative colitis and suppress the production of IL-6 in colonic tissues. Recent studies have also revealed antioxidant properties of indirubin that could counteract oxidative stress in colitis [27]. 

Taken together, enhanced dissolution and absorption of indirubin facilitated by microemulsion formation in the GI tract may be associated with the attenuation of inflammatory symptoms following oral administration of the SMEDDS-F1 formulation. Given that (i) MT-102 is an herbal extract including multiple active ingredients, and (ii) SMEDDS-F1 may improve the dissolution of other active and auxiliary substances, SMEDD-F1 may be advantageous for achieving more synergistic effects in therapeutic efficacy compared to isolated indirubin. Xie et al. [28] also support the synergistic effect of multiple components in treating ulcerative colitis. They showed that combined use of indirubin and indigo was more effective in ameliorating DSS-induced colitis by improving the intestinal barrier function compared to indirubin or indigo alone.

## 4. Conclusions

A self-microemulsifying drug delivery system of MT-102 (SMEDDS-F1) was successfully formulated using Capmul MCM, Tween 80, and propylene glycol as the ternary phase components. The optimized formulation of SMEDDS-F1 displayed elevated indirubin content and a restricted particle size distribution, with an average particle size of 177.5 ± 2.80 nm. Notably, SMEDDS-F1 significantly enhanced both the rate and extent of indirubin release from MT-102 across a pH range from acidic to neutral, with dissolution rates approximately 15-fold higher than pure MT-102. It also demonstrated similarly high indirubin release in both FeSSIF and FaSSIF, suggesting the minimal impact of food intake on the drug absorption in the GI tract. Furthermore, oral administration of SMEDDS-F1 demonstrated effectiveness in mitigating the symptoms of ulcerative colitis and inhibiting pro-inflammatory cytokine production, whereas the therapeutic effect of unmodified MT-102 was limited. SMEDDS-F1 minimized body weight loss without any significant change in colon length and the morphology of colonic tissues compared to those in the healthy control group. Collectively, these findings indicate that the developed SMEDDS-F1 has the potential to enhance the therapeutic efficacy of the poorly soluble MT-102.

## Figures and Tables

**Table 1:** Emulsification efficiency and percentage transmittance of various surfactants and cosurfactants (mean ± SD, *n* = 3).

	No. of Inversions	% Transmittance
Surfactants		
Labrafil 1944 CS	36	29.53 ± 3.21
Labrafil 2125 CS	28	32.78 ± 1.60
Kolliphor ELP	29	98.64 ± 0.51
Kolliphor RH40	21	99.37 ± 0.10
Labrasol	10	20.04 ± 0.77
Kolliphor HS 15	12	92.21± 6.99
Tween 80	11	99.63 ± 0.61
Cosurfactants		
Ethanol	11	99.85 ± 0.20
PEG 200	18	100.01 ± 0.41
Propylene Glycol	14	99.25 ± 0.24
Transcutol HP	11	99.40 ± 0.81
Capmul PG8	38	95.80 ± 1.60

**Table 2:** Characteristics of MT-102-loaded SMEDDS (SMEDDS-F1) (mean ± SD, *n* = 3).

Ratio (*w*/*w*)		Size (nm)	PDI	Zeta-Potential (mV)	Indirubin Content (µg/g)	Emulsification Time (s)	Transmittance (%)
Oil	S_mix_ (1:1)						
15	85	177.5 ± 2.80	0.24 ± 0.01	−7.34 ± 0.33	275.21 ± 5.58	27	90.56 ± 0.021

**Table 3:** Thermodynamic stability of SMEDDS-F1 under stress conditions.

Treatment	Clarity *	Size (nm)	PDI	Zeta Potential (mV)	Indirubin Content (µg/g)
Centrifugation	Clear	176.6 ± 6.14	0.21 ± 0.01	−7.41 ± 0.47	267.62 ± 12.99
Heat and cool cycle	Clear	190.6 ± 3.51	0.21 ± 0.01	−8.26 ± 0.41	261.86 ± 4.61
Freeze–thaw cycle	Clear	171.3 ± 3.93	0.22 ± 0.02	−8.22 ± 0.26	238.96 ± 14.53

* There is no phase separation, precipitation, or cloudiness.

**Table 4:** Robustness to dilution of SMEDDS-F1.

	Distilled Water			0.1 N HCl			Phosphate Buffer (pH 6.8)		
Dilution Fold	10	100	1000	10	100	1000	10	100	1000
Clarity	Clear	Clear	Clear	Clear	Clear	Clear	Clear	Clear	Clear
Size (nm)	191.9 ± 2.44	169.2 ± 1.14	156.1 ± 6.62	190.1 ± 0.90	168.8 ± 4.09	165.4 ± 3.16	181.2 ± 0.49	170.4 ± 2.65	159.0 ± 0.67

## Data Availability

Data are contained within the article and Supplementary Materials.

## Supplementary Materials

The following supporting information can be downloaded at: https://www.mdpi.com/article/10.3390/pharmaceutics15122720/s1, Table S1: Characteristics of drug-loaded SMEDDS at the different ratios of oil and S_mix_ (mean ± SD, n=3).
