# Supplementary material for: Improved Therapeutic Efficacy of MT102, a New Anti-Inflammatory Agent, via a Self-Microemulsifying Drug Delivery System, in Ulcerative Colitis Mice
Source: Pharmaceutics. 2023 Dec 2;15(12):2720. doi: 10.3390/pharmaceutics15122720 (PMC10747691; doi:10.3390/pharmaceutics15122720)
Supplement: Supplementary file 1 [file pharmaceutics-15-02720-s001.zip › pharmaceutics-2726559-supplementary.pdf]

pharmaceutics

Supplementary Material: Improved Therapeutic Efficacy of MT102, a New Anti-Inflammatory Agent, via a Self-Microemulsifying Drug Delivery System in Ulcerative Colitis Mice

Kshitis Chandra Baral, Sang Hoon Lee, Jae Geun Song, Seong Hoon Jeong and Hyo-Kyung Han

Table S1. Characteristics of drug-loaded SMEDDS at the different ratios of oil and S_{mix} (mean ± SD, n=3).

Ratio (w/w)		Size (nm)	PDI	Zeta Potential (mV)	Indirubin content (μg/g)	Emulsification time (Sec.)	% Transmittance
Oil	S_{mix}*						
10	90	118.7± 6.32	0.41± 0.08	-7.87 ± 0.36	242.84± 6.56	31	85.03± 0.67
20	80	249.5± 4.08	0.27± 0.02	-7.62 ± 0.54	193.21± 14.91	22	79.81± 0.67

*S_{mix}: Tween 80/propylene glycol = 1:1 (w/w).
